# Management of retinoblastoma with extraocular tumour extension

**Published:** 2018-06-03

**Authors:** Swathi Kaliki, Vijay Anand Reddy Palkonda

**Affiliations:** 1 Head of The Operation Eyesight Universal Institute for Eye Cancer: LV Prasad Eye Institute, Hyderabad, India.; 2 Consultant Radiation Oncologist: L V Prasad Eye Institute & Senior Consultant, Professor and Head: Department of Radiation Oncology, Director: Apollo Cancer Hospital, Hyderabad, India.

**Survival rates in children with extraocular tumour extension can be improved with a combination of chemotherapy, surgery, and radiotherapy.**

**Figure:**
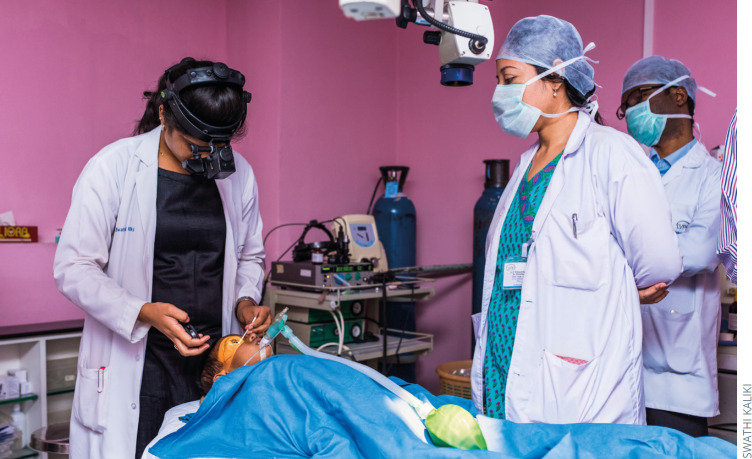
Enucleation is just one step in treating extraocular retinoblastoma. INDIA

In high-income countries, the survival rate of children with retinoblastoma has improved over the years: from 5% to >95%. This improvement is a result of early diagnosis and intervention by specialist retinoblastoma teams. However, in low- and middle-income countries, the survival rate continues to remain low, with 39% mortality rates in Asia and 70% in Africa.^1^ The high death rate in low- and middle-income countries is mainly related to the delay between onset of symptoms and start of treatment. This is due to a combination of factors: poor access to health care services, poor socioeconomic conditions and poor education; resulting in advanced disease at presentation. Added to this is poor compliance with treatment; in particular, refusal for potentially life-saving enucleation due to cultural beliefs.

While leucocoria (a white reflex) is a common presenting sign of retinoblastoma in most high-income countries, an enlarged eye or proptosis with extraocular tumour extension remains the most common presenting sign in low- and middle-income countries.^2^ It was previously estimated that only 9% of patients with orbital extension of retinoblastoma (stage III disease: see page 11) live for more than two years after diagnosis of retinoblastoma. However, recent literature suggests that, with a combination of chemotherapy, surgery and radiotherapy, the five-year survival rate of patients with stage III disease (orbital extension of retinoblastoma) is >50%, while the prognosis for stage IV disease with systemic metastasis or central nervous system involvement still remains dismal.^3^

## Recommended treatment for retinoblastoma with extraocular tumour extension

### Assessment of disease spread

Perform a general examination and examine both eyes thoroughly, preferably under anaesthesia. Since bone marrow and cerebrospinal fluid are two potential sites for tumour spread, bone marrow aspiration and cerebrospinal fluid analysis are recommended before starting treatment. If there is enlargement of regional lymph nodes, a fine needle aspiration biopsy can be undertaken to determine if the tumour has spread to the nodes. A magnetic resonance imaging or computed tomography of the orbit should be done to ascertain the extent of the disease.

### Treatment

**Figure 1:**
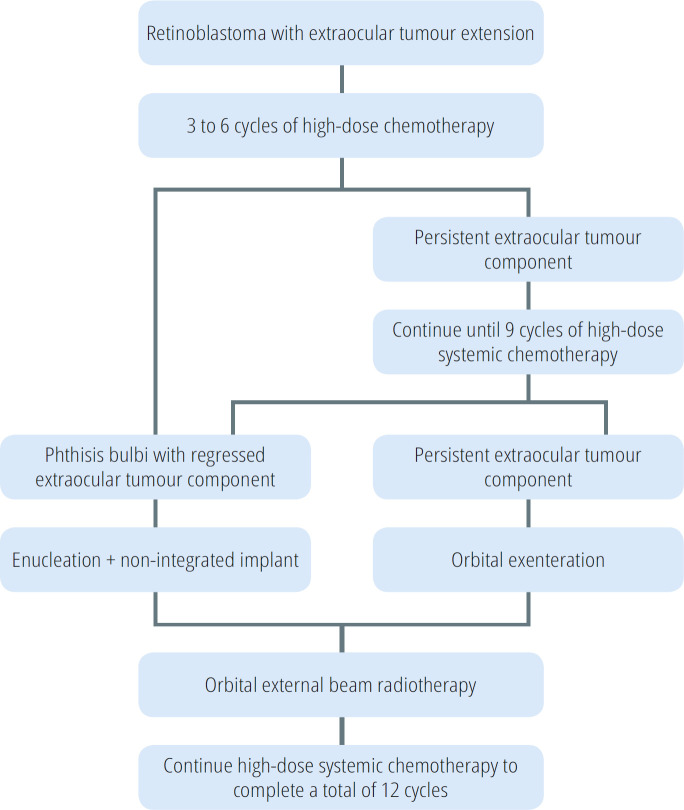
Line diagram depicting the recommended protocol for retinoblastoma with extraocular extension

In this article, we describe the treatment protocol we have used in our clinic since the year 2000. Treatment includes several cycles of high-dose systemic chemotherapy, followed by surgery (enucleation or exenteration), and then external beam radiotherapy to the orbit. Whenever there is an overt extraocular tumour extension, including gross optic nerve extension or extrascleral tumour extension, high-dose systemic chemotherapy should be given every 3 weeks as the primary treatment. Primary enucleation or orbital exenteration should be avoided until after the tumour shrinks so that surgery is more successful without leaving behind tumour residue. Various combinations of chemotherapy have been reported in the literature. We currently use the combination of vincristine, etoposide, and carboplatin, and have found it to be effective (Table 1, Figure 1).

**Table 1:** High-dose systemic chemotherapy for retinoblastoma with extraocular extension

Drug	Dose	
	Children <3 years	For children >3 years
Vincristine	0.05 mg/kg	1.5 mg/m^2^ body surface area (BSA)
Etoposide	10 to 12 mg/kg	200 mg/m^2^ BSA
Carboplatin	28 mg/kg	560 mg/m^2^ BSA
**Regimen of each cycle**	**Drugs delivered**	
Day 1	Vincristine + Etoposide + Carboplatin	
Day 2	Etoposide	

**Figure 2:**
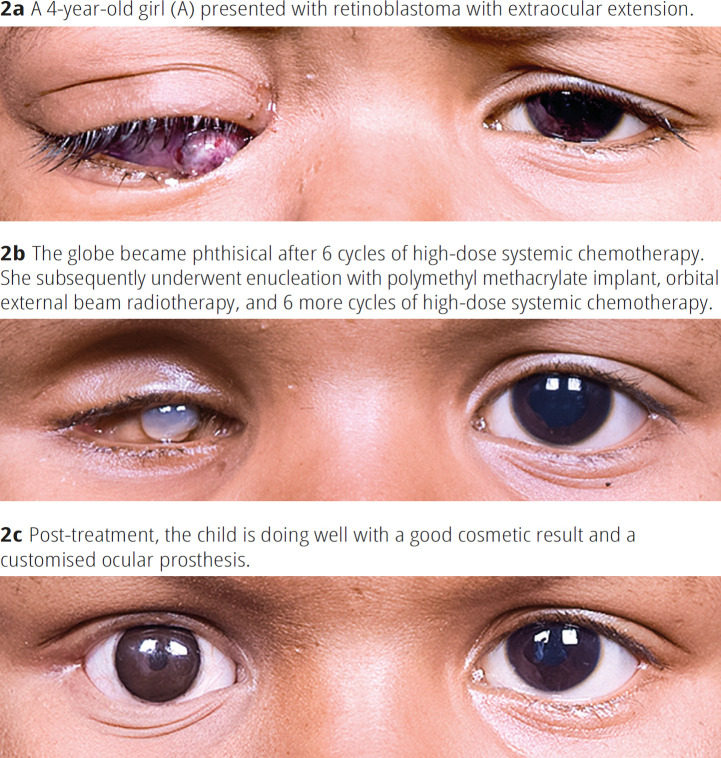
Treatment of a retinoblastoma patient with extraocular extension

High-dose systemic chemotherapy is given until the extraocular component of the tumour regresses. An average of 6 cycles of high-dose systemic chemotherapy results in complete regression of the extraocular tumour component in 95% cases, thus avoiding the need for orbital exenteration (Figure 2). Repeat magnetic resonance imaging or computed tomography of the orbit can be used to determine regression or persistence of extraocular tumour.

In eyes with regressed extraocular tumour, secondary enucleation with placement of an implant is performed. In eyes with residual extraocular tumour despite a maximum of 9 cycles of high-dose systemic chemotherapy, secondary orbital exenteration is recommended.

Six weeks after surgery, external beam radiation (45 to 50 Gy) is delivered to the orbit. The radiation field should include the regional lymph nodes if the patient had regional lymph node involvement at presentation.

Post-radiation, high-dose systemic chemotherapy is continued to complete a total of 12 doses of chemotherapy. In cases with stage IV disease at presentation with central nervous system involvement, additional intrathecal chemotherapy is recommended.

Using this treatment protocol, in our recent analysis of 20 patients with stage III disease who were compliant with treatment, 17 patients survived and were doing well at a median follow-up duration of 77 months.^3^ Three patients died despite adherence to treatment protocol. All patients who were noncompliant to treatment eventually died due to the disease. All patients with stage IV disease died despite aggressive multimodal treatment.

In summary, early diagnosis and appropriate treatment of retinoblastoma is crucial to improve the chances of life, globe, and vision salvage. In patients with delayed presentation including extraocular extension of retinoblastoma but without systemic or central nervous system metastases, multimodal treatment in specialist centres improves the chances of survival. The compliance to treatment plays a very important role in determining the likelihood of survival.
